# Immune evasion strategies of neuroendocrine-like Enzalutamide resistant prostate cancer

**DOI:** 10.1186/2051-1426-1-S1-P147

**Published:** 2013-11-07

**Authors:** Jennifer L Bishop, Barinder Sangha, Martin Gleave, Amina Zoubeidi

**Affiliations:** 1 Urologic Sciences, University of British Columbia, Vancouver, BC, Canada; 2 Vancouver Prostate Centre, Vancouver, BC, Canada; 3 Molecular Biology and Biochemistry, Simon Fraser University, Burnaby, BC, Canada

## 

Treatment with Enzalutamide (ENZ) prolongs survival in patients with incurable castration-resistant prostate cancer (CRPC). However, the rapid recurrence of ENZ resistant tumors and the increased incidence of lethal neuroendocrine cancers in CRPC patients underscore the need for understanding how aggressive cell types emerge in resistant disease in order to design more effective therapies. As an unbiased approach to uncovering novel mechanisms of ENZ resistance, ENZ resistant cells were established from castration-resistant LNCaP xenografts that recurred under ENZ treatment in vivo and were genetically profiled using Illumina Platform gene expression analysis as well as RNA Seq. Genes involved in myeloid cell differentiation and immune checkpoint responses, as well as neuroendocrine differentiation, were strongly upregulated in ENZ resistant cell lines compared to CRPC controls. To investigate whether ENZ resistant cells may pass through a “dedifferentiated” state allowing progression to a neuroendocrine phenotype with immune-evasive properties, markers of cancer stem cells (CD133, CD44, CD117, CD49F, TROP2) neuroendocrine cells (neuron specific enolase, snaptophysin, chromagranin A, aurora kinase A, NCAM-1) and immune-evasion (programmed death ligands-1 and 2, B7H3, ICOSL) were assessed by quantitative PCR and flow cytometry. Indeed, all cancer stem cell markers were expressed at a higher frequency on ENZ resistant cells than CRPC controls and neuroendocrine markers were highly upregulated. Consistent with gene profiling results suggestive of a myeloid differentiation process, neuroendocrine-like resistant cells displayed an “immune” phenotype, expressing cell surface markers and transcription factors characteristic of dendritic cells (CD11c, HLA-DR, IRF8, ID2, AHR, BATF) that were absent from CRPC cells. Moreover, a greater frequency of neuroendocrine-like ENZ resistant cells expressed PD-L1, PD-L2 and ICOSL, and they showed intense staining for B7H3. Each of these molecules is well known to limit T cell effector responses and have been implicated in immune evasion strategies of solid tumors. Taken together, our results suggest that acquisition of resistance to Enzalutamide promotes the emergence of neuroendocrine-like cells that may be able to subvert anti-tumor T cell responses via dysregulation of antigen presentation or by enhancing immune checkpoint signaling. These results support anecdotal findings of increased frequency of neuroendocrine prostate cancer in patients undergoing long-term androgen withdrawal therapy and suggest investigating the utility of immunotherapies, such as anti-CTLA-4 or PD-1, in this subset of patients.

